# Aqueous synthesis of LiFePO_4_ with Fractal Granularity

**DOI:** 10.1038/srep27024

**Published:** 2016-06-03

**Authors:** Zahilia Cabán-Huertas, Omar Ayyad, Deepak P. Dubal, Pedro Gómez-Romero

**Affiliations:** 1 Catalan Institute of Nanoscience and Nanotechnology (ICN2), CSIC and The Barcelona Institute of Science and Technology, Campus UAB, Bellaterra, 08193 Barcelona, Spain

## Abstract

Lithium iron phosphate (LiFePO_4_) electrodes with fractal granularity are reported. They were made from a starting material prepared in water by a low cost, easy and environmentally friendly hydrothermal method, thus avoiding the use of organic solvents. Our method leads to pure olivine phase, free of the impurities commonly found after other water-based syntheses. The fractal structures consisted of nanoparticles grown into larger micro-sized formations which in turn agglomerate leading to high tap density electrodes, which is beneficial for energy density. These intricate structures could be easily and effectively coated with a thin and uniform carbon layer for increased conductivity, as it is well established for simpler microstructures. Materials and electrodes were studied by means of XRD, SEM, TEM, SAED, XPS, Raman and TGA. Last but not least, lithium transport through fractal LiFePO_4_ electrodes was investigated based upon fractal theory. These water-made fractal electrodes lead to high-performance lithium cells (even at high rates) tested by CV and galvanostatic charge-discharge, their performance is comparable to state of the art (but less environmentally friendly) electrodes.

Lithium ion batteries (LIBs) have revolutionized portable electronic devices in the past two decades, and are primed to make a great impact on transportation technology by powering electric vehicles (EVs). However, this new application demands that LIBs offer higher energy and higher power capabilities at a lower cost and with environmentally friendly materials^1^. Since the seminal work of Goodenough and col.^2^, lithium iron phosphate (LiFePO_4_) has been researched as cathode material for LIBs thanks to its low cost, abundant raw materials, safety, low toxicity, structural stability and excellent electrochemical properties. The active material can be reversibly charged and discharged with a stable voltage profile at 3.45 V vs. Li^+^/Li with a very small change in unit cell parameters during the LiFePO_4_/FePO_4_ phase transition. On the other hand, for the development of high power batteries based on this material, it is essential to understand and overcome the factors limiting lithium transport through the electrode. Indeed, despite its high theoretical specific capacity (170 mAh/g) and long cycling lifetime, the high-rate performance of the raw LiFePO_4_ is restricted by its poor electronic conductivity (10^−9^ S/cm) and slow lithium diffusion^3^. Many different approaches involving surface coating have been tried to improve the capacity and rate performance of LiFePO_4_ as cathode for LIBs. Increasing the conductivity by coating the LiFePO_4_ surface with carbon^4^ or conducting polymers^5^,^6^ have been two of the most popular.

In addition to coating, the control of surface microstructure constitutes another general approach towards faster electrode reaction for batteries. Among many possible alternatives, fractal electrode design is proposed as a very promising approach for high-performance batteries, since it greatly improves the surface to volume ratio while providing a high energy-density material with large tap density. Thus, the growth of self-assembled nanoparticles into larger microstructures can provide high surface area for high power and large bulk for high energy density. In addition, mass transfer in LIBs can be improved by spreading the reaction sites throughout the entire volume of the device. Space filling fractal networks can work to ensure that there is efficient charge transfer from a huge effective surface area to a current collector. Last but not the least; a fractal granular microstructure could minimize the internal resistance of the electrode. Recently, micro-sized yet porous LiFePO_4_ structures have been reported with high electronic conductivity and fast Li^+^ permeation. For instance, Liu *et al.* reported the synthesis of 3D nanoporous spherical LiFePO_4_/C material by spray pyrolysis techniques^7^. The 3D conductive carbon coating with interconnected pore networks facilitate both electron transport as well as lithium ion diffusion within the particles, leading to excellent cycling performance and rate capability^7^. However, most of the techniques used to grow fractal structures are based on organic solvents, surfactants and templates which make them less attractive considering production cost of the materials^8^. Therefore the development of effective routes for the synthesis of fractal micro-structured LiFePO_4_/C at an ever lower cost still represents a great challenge.

Herein, we are introducing an example of fractal granular LiFePO_4_ electrode with excellent electrochemical properties. Briefly, a cost-effective, water-based hydrothermal method is used to prepare micro-nano structured fractal LiFePO_4_ materials and electrodes. The samples were fully characterized with different physical-chemical techniques to provide proof of concept. Furthermore, the present work is aimed to investigate the boundary conditions at the electrode surface for lithium transport, and the effect of surface roughness on the diffusion-controlled lithium transport. The surface morphology of the electrodes was examined by AFM and their apparent self-similar dimensions were determined by a triangulation method.

## Results

**Figure 1:**
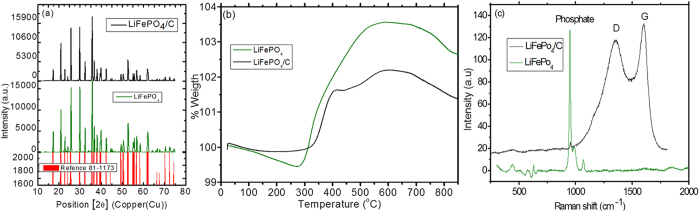
(**a**) XRD patterns of pristine LiFePO_4_ and LiFePO_4_/C samples, (**b**) TGA curves of LiFePO_4_ and LiFePO_4_/C, (**c**) Raman spectrum of the LiFePO_4_/C.

Figure 1(a) shows XRD patterns of pristine LiFePO_4_ and LiFePO_4_/C samples. All diffraction peaks are indexed to orthorhombic LiFePO_4_ (JCPDS card number 081-1173, space group Pnma). It is very important to note that no impurities are detected. This is in contrast with some previous reports on solvothermal syntheses making use of water or organic solvents leading to detrimental impurities^9^. Some researchers reported impurities such as iron phosphides Fe_2_P^10^ which often form at high temperatures (>600 °C)^11^ or LiFe(P_2_O_7_)^12^, Fe (II, III) pyrophosphates or phosphates Li_3_Fe_2_(PO_4_)_3_^13^ and Li_3_PO_4_^14^. This lack of impurities confirms the suitability of our water-based hydrothermal method for the successful synthesis of pure stoichiometric LiFePO_4_ material. Moreover, all diffraction peaks are intense and narrow for our samples, indicating a high degree of crystallinity of the LiFePO_4_ phase prepared both before and after carbon-coating.

Figure 1(b) shows TGA curves of pristine LiFePO_4_ and LiFePO_4_/C composite under flowing air atmosphere. These experiments were carried out to study the thermal stability of the materials and to determine the exact amount of carbon coated on LiFePO_4_ active phase. After an initial weight loss associated to loss of water, the TGA curves show a weight gain of 5.0% for LiFePO_4_ and 2.4% for LiFePO_4_/C between 250–650 °C. The weight uptake of pristine LiFePO_4_ can be explained by the following oxidation reaction^15^:


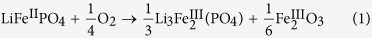


The amount of carbon coated on LiFePO_4_ was calculated by measuring the difference between the total weight gain of LiFePO_4_ and that of LiFePO_4_/C and turned out to be 2.6%.

The LiFePO_4_/C sample was also analyzed by Raman spectroscopy in order to investigate the nature of coated carbon. As seen in Fig. 1(c), two intense broad peaks were recorded at 1330 cm^−1^ and 1595 cm^−1^ corresponding to the A_1__g_ vibration mode of the disordered carbon (D-band) and E_2__g_ vibration mode of the ordered graphitic carbon (G-band), respectively^16^. The ratio of intensities of D-band to G-band (I_D_/I_G_) is 0.87, indicating sp^2^ carbon, which would enhance the electronic conductivity of the LiFePO_4_ material^17^.

**Figure 2:**
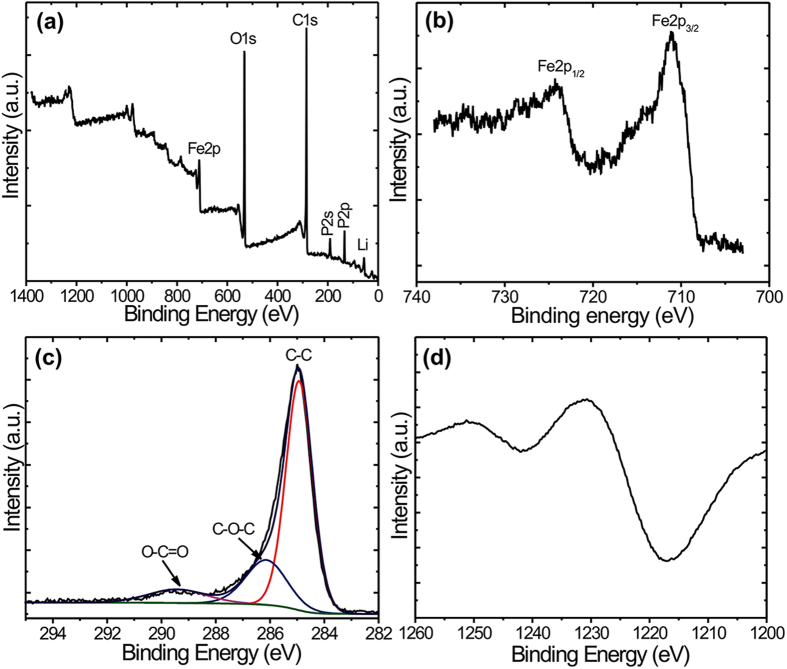
XPS spectra of the LiFePO_4_/C powder calcined at 700 °C (**a**) Full spectrum of LiFePO_4_/C (**b**) narrow spectrum of Fe_2_p (**c**) core-level XPS spectrum of C1s and (**d**) Derivate of the carbon auger peak.

The chemical composition and valence state of LiFePO_4_/C material was confirmed by XPS analysis. Figure 2(a) shows the wide range-scanning spectrum, which consists of Li, Fe, P, O and C components confirming formation of LiFePO_4_/C material. The Fe_2_p spectrum (Fig. 2(b)) exhibits two major peaks (Fe 2p_3/2_ and Fe 2p_1/2_) at binding energies of 710.9 eV and 724.2 eV indicating Fe(II) valence state which is characteristic of the olivine-type LiFePO_4_ products^18^. Figure 2(c) shows the deconvolution of the C 1 s spectrum in LiFePO_4_/C, clearly displaying the lower binding energy featured at 284.6 eV corresponding to C-C carbon and the higher binding energy featured at 286.1 eV followed by a shoulder at 288.9 eV, which was typically assigned to C-O-C, O-C = O arising from epoxide, carboxyl functionalities^19^. Figure 2(d) shows the fine structure of C KLL transition, which is strongly affected by the sp2/sp3 configuration. Parameter *D* can be considered as a fingerprint of the type of carbon hybridization, showing values of 13.7 eV for sp2 and 21.2 eV for sp3 hybridization states^20^. For extended carbon phases the D value can therefore represent a diagnose of the conducting character of a given material since sp2 is associated to graphitic carbons, better conducting than sp3 structures. The D parameter value for carbon in our LiFePO_4_/C sample turned out to be 16.2 eV which indicates an intermediate composition of sp2 and sp3, corresponding to a mixture of ca. 2/3 sp3 and 1/3 sp2 C in the sample. This means that although the carbon coating of LiFePO_4_ is not purely sp2 it does contain enough sp2 carbon to provide electrical conductivity of the material.

**Figure 3:**
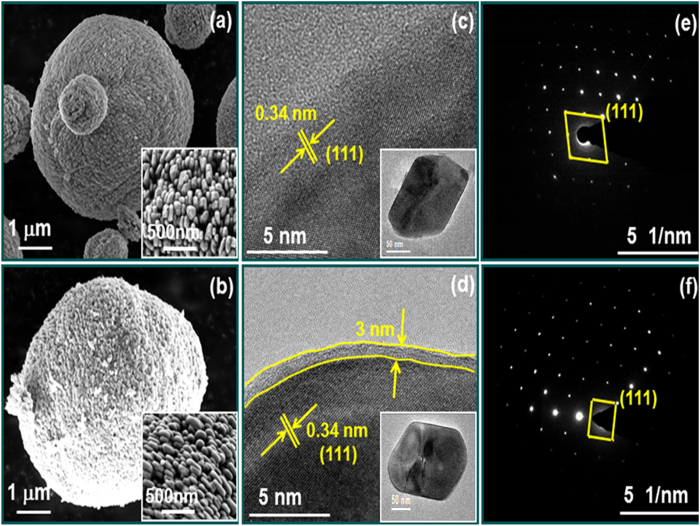
(**a,b**) SEM images of LiFePO_4_, LiFePO_4_/C, inset shows high magnified images. For these two images isolated spheres were selected. Most abundant agglomerates of these secondary spherical particles are shown in Supporting Information 2. (**c,d**) HR-TEM images of LiFePO_4_, LiFePO_4_/C, inset shows low magnified images (**e,f**) SAED patterns of LiFePO_4_, LiFePO_4_/C, respectively.

**Figure 4:**
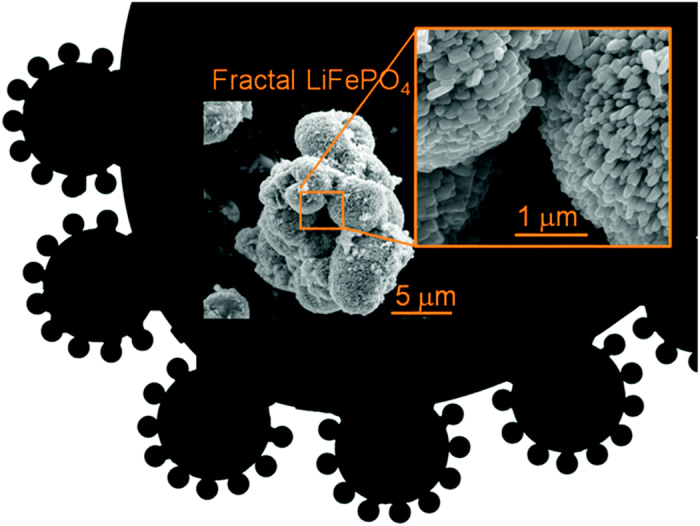
Scheme of LiFePO_4_ fractal granularity design.

The microstructures of the LiFePO_4_ and LiFePO_4_/C samples were investigated by SEM and are presented in Fig. 3(a,b). As seen in Fig. 3(a), our LiFePO_4_ sample is constituted of large microspheres a few microns in diameter formed in turn by a very large number of nanoparticles of ca. 200 nm in a configuration, which could be described as fractal. Indeed, nanosized primary particles conformed the surface of microsized secondary particles form a microstructure reminiscent of the black fractal sketch shown in the Fig. 4. This configuration presents the advantages of nanoparticulate matter and the easy handling and high tap density of microparticles. Inset of Fig. 3(a,b) show details of the closely packed primary nanoparticles featuring inter-particle slit pores. Comparing both of them allows us to conclude that this fractal granularity is retained after the pyrolysis treatment for carbon-coating (Fig. 3b). These results are in contrast to previously reported conventional hydrothermal synthesis of LiFePO_4_ which led to very large microcrystals (in air) and required N_2_ atmosphere for further growth of smaller particles^21^. In the present work, LiFePO_4_ fractal granular geometry is achieved with the simple addition of polyethyleneimine (PEI), which controls the growth of these optimal nano-microstructures without any special treatment.

The samples were also studied with TEM. Figure 3(c,d) compares high-resolution images of pristine and carbon-coated LiFePO_4_ primary nanocrystals. From the HRTEM image (Fig. 3c,d), one can clearly see the lattice fringes with an interplanar spacing of 0.34 nm for both LiFePO_4_ and LiFePO_4_/C nano-particles, which is identified as the characteristic interplanar spacing of the (111) plane of olivine-type LiFePO_4_ material. The carbon layer covering the LiFePO_4_ surface has an average thickness of 3 nm and is clearly observed in Fig. 3(d). No long-range order is apparent in this carbon layer; yet it must be composed of conducting graphitic carbon (sp2 according to Raman) comparable to graphene domains (but not diffracting due to its small thickness). Insets of Fig. 3(c,d) show low-magnification TEM images of LiFePO_4_ and LiFePO_4_/C single crystals, confirming identical nanoparticle morphologies. Moreover, the selected area electron diffraction (SAED) patterns of both LiFePO_4_ and LiFePO_4_/C (Fig. 3e,f) were indexed as (111) plane confirming the LiFePO_4_ orthorhombic structure in agreement with XRD results.

The fractal granularity of LiFePO_4_ leads to a characteristic slit porosity formed by the primary nanosized crystals (better seen in the inset of Fig. 3(a,b). These inter-particle pores extend from the surface to the inner core of the spheres and can facilitate deep penetration of liquid electrolyte solution into the microspheres, thus providing an improved interface contact between the electrode and electrolyte. (supporting information S1).

**Figure 5:**
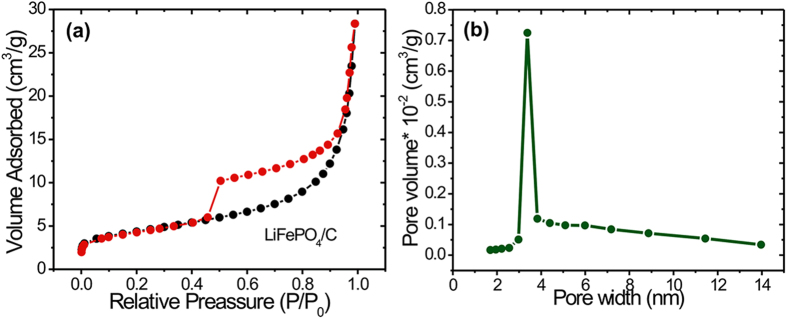
(**a**) Nitrogen adsorption/desorption isotherms for LiFePO_4_/C (**b**) Barret-Joyner-Halenda (BJH) pore size distribution curve for LiFePO_4_/C.

To further investigate the surface properties of fractal LiFePO_4_ material, we performed Brunnauer-Emmett-Teller (BET) analysis of adsorption isotherms shown in Fig. 5(a). The LiFePO_4_/C sample shows a typical isotherm of type IV with hysteresis loop in a relative pressure (*p/p*_*0*_) range of 0.4–1.0, implying the formation of slit-like pores^22^, a type of porosity which can be easily understood as a result of the stacking of nanoparticles. The measured BET surface area was found to be 14.8 m^2^/g for LiFePO_4_/C. Figure 5(b) shows the Barett-Joyner-Halenda (BJH) pore size distribution curve with a distinct maximum centered at ~3.7 nm. This confirms the mesoporous nature of the LiFePO_4_/C fractal structure. The mesoporosity of LiFePO_4_/C samples results from a combination of internal space of the agglomerated nanoparticles and microspheres. Despite the modest value of the BET surface area, such a mesoporous structure facilitates diffusion of Li ions from the electrolyte into the electrode bulk by providing short diffusion lengths.

The microstructural characterization of LiFePO_4_ was completed with two more complementary techniques, namely AFM (20 × 20 microns) for electrode area analyses and SEM for cross-section (20 microns) analyses. Thus, cross-section SEM images of LiFePO_4_ and LiFePO_4_/C electrodes coated on Al substrates with elemental composition analyses were carried out and are shown and discussed in supporting information S2 and S3. The film thickness was between 15 μm and 20 μm for LiFePO_4_ and 5–10 μm for LiFePO_4_/C.

**Figure 6:**
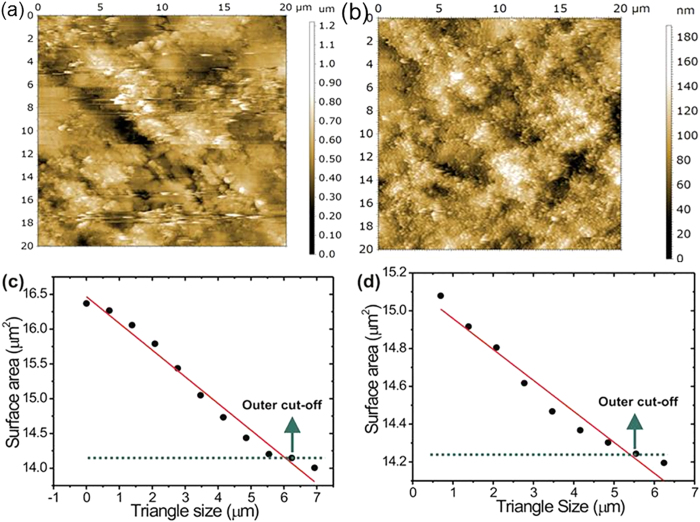
(**a,b**) AFM images of LiFePO_4_ and LiFePO_4_/C electrodes, respectively (**c,d**) Dependence of surface area SA on triangle size TS obtained from AFM images of LiFePO_4_ and LiFePO_4_/C electrode, respectively. The slope s means (d log SSA/d log TS).

In order to get a better understanding of Li transport and confirm the fractal nature of LiFePO_4_, surface roughness was analyzed in triangles of various sizes for a total area of 20 × 20 microns by AFM. Figure 6(a,b) shows AFM surfaces of LiFePO_4_ and LiFePO_4_/C film electrodes, respectively. It can be noted that the LiFePO_4_/C film electrode shows less roughness than LiFePO_4_ film electrode. In order to get quantitative insights, root mean square (rms) roughness of both electrodes was evaluated (Table 1). Since these structures appear to be fairly similar on various length scales, this surface can be regarded as a self-similar fractal. The triangulation method, reported elsewhere^23^, was used to determine the self-similar fractal dimensions. Figure 6(c,d) gives logarithmic scale dependence of scaled surface area (SSA) on projected triangle size (TS) obtained from the LiFePO_4_ and LiFePO_4_/C film electrodes, respectively.

For both electrodes, one can find clearly a linear relationship between the log(SSA) and log(TS), indicating the self-similar scaling property of the surface. It is generally known that the self-similar fractal dimension Df, ss of a surface is given in terms of the slope of the straight line ‘s’ (=d log SSA/d log TS) as^24^


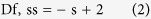


From Fig. 6, the outer cut-offs for their fractal structures were found to be 6.5 and 5.5 μm, respectively. Here, it should be stressed that although both LiFePO_4_ film electrodes show the self-similar scaling properties, the spatial outer cut-offs for their fractal structures are different from each other.

**Figure 7:**
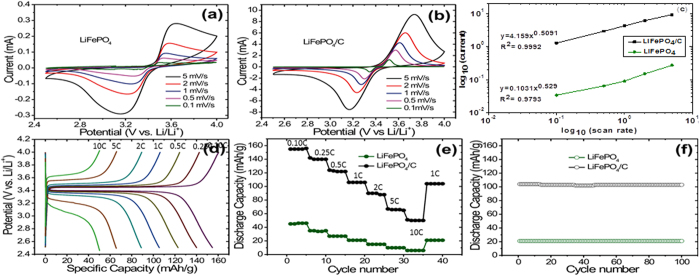
(**a,b**) Cyclic voltammetry (CV) curves for LiFePO_4_ and LiFePO_4_/C electrodes at different scan rates, respectively (**c**) Dependence of anodic peak current I_peak_ on scan rate for LiFePO_4_ and LiFePO_4_/C electrodes with logarithmic scale (**d**) Charge/discharge curves at various C-rates for LiFePO_4_/C (5^th^ cycle) (**e**) Discharge capacity at different C-rates of LiFePO_4_ and LiFePO_4_/C (**f**) Cycling performance of pristine LiFePO_4_ and LiFePO_4_/C at 1C.

The electrochemical properties of LiFePO_4_ and LiFePO_4_/C electrodes were further investigated, results presented in Fig. 7. The ionic diffusion in the self-similar fractal electrode was studied by CV. The sharp oxidation and reduction peaks in CV curves for LiFePO_4_/C electrode (see Fig. 7a,b) confirm the excellent reversibility of the Li extraction-insertion reaction as compared to that for pristine LiFePO_4_ electrode. The well-defined oxidation and reduction peaks at ca. 3.4 V and 3.6 V are assigned to the Fe^2+^/Fe^3+^ redox couple, corresponding to the Li insertion (Fe^3+^ to Fe^2+^) and extraction (Fe^2+^ to Fe^3+^) in the LiFePO_4_ crystal structure, respectively. In contrast, the CV curve recorded for pristine LiFePO_4_ is broader and exhibits relatively low current density. This may be attributed to low conductivity and slow Li diffusion in pristine LiFePO_4_ electrode.

Strømme *et al.* suggested the peak current method to determine the fractal dimension of a given electrode surface by using cyclic voltammetry: when the recorded cyclic voltammetry current is limited by diffusion of the electroactive species and away from the electrode surface, the fractal dimension *d*_*f*_ of the reaction site on the surface can be obtained by the following equation,


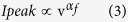


where


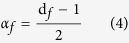


And *I*_*peak*_ is the intensity of the reduction peak, and **υ** is the scan rate. The slope of the plot log(*Ipeak*) vs log (v) is α_f_ and is called the fractal parameter, which is related to the fractal dimension of the surface as indicated in (4)

Figure 7(a–c) is also used to provide information of the ion diffusion coefficient using an improved Randles–Sevcik equation (see Supplementary Information S4) applicable to fractal electrodes^25^


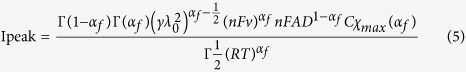


where *I*_*peak*_ is the peak current, ‘Γ’ the gamma function, ‘γ’ a geometrical factor close to π^−1^, λ_0_ the length corresponding to the outer cut-off of the fractal electrode, ‘n’ the number of transferred electrons per atom, ‘F’ Faraday’s constant, ‘*v*’ the scan rate, C the concentration of the electrolyte, χ_max_ a dimensionless function of the fractal parameter given in ref. 26, R the gas constant, and T the temperature. ‘n’ is equal to 1 since the reaction is


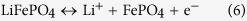


According to equation 4, the diffusion coefficients were found to be 4.11 × 10^−14^ cm^2^/s for LiFePO_4_ and 1.83 × 10^−13^ cm^2^/s for LiFePO_4_/C, comparable to previous values found in the literature (6.56 × 10^−16^ to 0.52 × 10^−12^)^27^,^28^, indicating considerable increases in diffusivity after modifications. This study examines the effects of a carbon coating on the electrochemical performances of LiFePO_4_. The effects of the carbon coating as well as the mechanisms for the improved electrochemical performances after modification are discussed based on the diffusivity data and confirm better diffusion in the C-coated sample. The results show that the capacity of bare LiFePO_4_ decreased sharply, whereas the LiFePO_4_/C shows a well maintained initial capacity.

Figure 7(d) shows the charge/discharge curves at various C rates from 0.1 C to 10 C for LiFePO_4_/C cathodes vs. lithium anodes. The LiFePO_4_/C exhibits a high specific charge value of 159 mAh/g (ca. 94% of the theoretical capacity of 170 mAh/g) at 0.10 C rate (charge and discharge). This charge at a discharge voltage of 3.4 V and a tap density of 1.70 Kg/L (see below) results in energy density of 920 Wh/L. In contrast, cathodes of the pristine LiFePO_4_ do not show a high specific discharge capacity. Even at 0.10 C rate the pristine LiFePO_4_ shows a discharge capacity of 45 mAh/g wich correspond to 27% of the theoretical capacity. Both samples show good stability at diferent C-rates (Fig. 7e). The discharge capacity of LiFePO_4_/C at 10 C correspond to 31% of the value obtained at 0.10 C, whereas for pristine LiFePO_4_ the discharge capacity at 10 C represents 22% of that at 0.10 C (Fig. 7f). The pristine LiFePO_4_ cathode material was stable during extensive cycling at 1 C; the capacity retention over 40 cycles is 99%, which is remarkable. Figure 7d shows the very good cyclability of LiFePO_4_/C cathode material. This sample was stable over 100 cycles with 98% retention of capacity.

The electrochemical results achieved in the present work are comparable to other reports in the literature, while improving the synthetic procedure with a low-cost water-based simple procedure. For example, Chen *et al*. reported a discharge capacity of 150 mAh/g at 0.1 C rate and 100 mAh/g at 1 C^22^ for LiFePO_4_/C, Yang *et al.* reported discharge capacity of 140 mAh/g for porous graphene/LiFePO_4_^29^ whereas Yu *et al.* prepared template-assisted porous LiFePO_4_ particles^30^ and reported capacity of 140 mAh/g at 0.10 C-rate.

## Discussion

In summary, we have developed a low-cost and eco-friendly hydrothermal synthesis of fractal granular LiFePO_4_ in aqueous media leading to a pure material, free of the impurities commonly associated to other water-based syntheses. This LiFePO_4_ material is composed of 200 nm nanocrystals grown into hierarchically superior spherical microstructures, in turn aggregated into larger units, thus featuring fractal granularity. The size of nanoparticles in fractal micro-structure is tuned by a small amount of polyethyleneimine (PEI) (5%), which plays a triple role as i) reducing agent, preventing oxidation of Fe(II), ii) surface modifier, limiting the growth of individual nanoparticles and iii) as polymer host acting as binder. Moreover, AFM analysis confirms that both electrodes have self-similar fractal nature, although they have different spatial outer cut-offs for their fractal granularity. Carbon coating is also easily and effectively attained without altering the initial fractal granularity of LiFePO_4_ electrode. As expected, carbon coating improves the conductivity of LiFePO_4_, hence its rate capability and cycling stability as LIB electrode is greatly enhanced. Furthermore, the fractal granularity provides high tap density while maintaining a highly dispersed active material at the nanometer level for an optimized electrode-electrolyte interphase.

## Methods

### Synthesis of LiFePO_4_ fractal design

Fractal LiFePO_4_ materials were synthesized through an optimized hydrothermal method. The starting materials were FeSO4.7H_2_O (99%, Sigma-Aldrich), H_3_PO_4_ (85%, Sigma-Aldrich) and CH_3_COOLi.2 H_2_O (reagent grade, Sigma-Aldrich) and were all used as received. PEI solutions in water (50 wt%) was also purchased from Sigma-Aldrich, this solution was pre-warmed to 70 °C for faster precursor dissolution. The molar ratio of Fe:P:Li was kept at 1:1:3. In a typical reaction, 6.6618 g of lithium acetate was added to 70 ml of a preheated 5% polyethylene imine solution in water and stirred for 10 minutes. 6.6175 g of iron sulfate was dissolved in 5% PEI (70 ml) and stirred for 2 minutes. Then, 1.65 ml of 85% phosphoric acid was slowly added to the iron sulfate solution, and the resulting solution was stirred for 10 minutes. Finally, both solutions (Li and Fe/PO_4_ solutions) were mixed together, stirred for 10 minutes and transferred into 200 ml hydrothermal reactor (Teflon vessel sealed in a stainless-steel autoclave). The reactor was maintained at 200 °C for 24 h. The resulting LiFePO_4_ pale green powder was filtered; washed with deionized water/ethanol and dried at 80 °C in a vacuum oven overnight. The final weight of the powder was 3.4932 g, which represents 93% of the theoretical yield.

In the next step, LiFePO_4_ particles were coated with carbon using glucose as carbon source. A solution of 10 wt% glucose and 90 wt% LiFePO_4_ in water was prepared. This solution was stirred for 5 hours, and then sonicated for 10 minutes. Later, the sample was filtered and dried overnight at 90 °C in a vacuum oven. The sample was initially calcined at 350 °C for 3 h and then sintered at 700 °C for 10 h, all under nitrogen atmosphere. The tap density of LiFePO_4_ samples was 1.40 g/cm^3^ (pristine) and 1.70 g/cm^3^ (carbon-coated)^31^,^32^, which are above the average tap-density values reported in the literature for LiFePO_4_^31^,^33^,^34^.

### Materials characterization

The phase purity and crystalline structure of the samples were determined by X-ray diffraction (XRD) by means of PANalytical X′Pert PRO diffractometer using a CuKα radiation source (λ = 1.5418 Å). The morphology of the samples was studied by scanning electron microscopy (SEM, FE Quanta 650 F ESEM) and transmission electron microscopy (TEM, Tecnai G2 F20 HRTEM) operated at an acceleration voltage of 200 keV. N_2_ adsorption/desorption was determined by Brunauer-Emmett-Teller (BET) measurements using Micromeritics instrument (Data Master V4.00Q, Serial#:2000/2400). Thermal gravimetric analysis (TGA 500Q) was carried out under air atmosphere between room temperature and 850 °C, and the flow rate of the synthetic air was 10 ml/min. Raman spectra were recorded on a HORIBA Scientific LabRAM HR Raman spectrometer system using Ar laser. The X-ray photoelectron spectra (XPS) analyses were obtained by X-ray photoelectron spectroscopy (XPS, SPECS Germany, PHOIBOS 150). Surface morphologies of the self-affine fractal electrodes were obtained with a SPA 400 equipped with a SPI3800N Probe Station (Seiko Instruments Inc.) in the atomic force microscope (AFM) mode using commercial silicon nitride cantilevers.

### Electrode preparation and electrochemical characterization

The cathodes were prepared by pressing a mixture of the active materials with carbon Super P (Timcal) and polyvinylidene fluoride (PVDF) binder in a weight ratio 85:10:5. They were mixed in a mortar for 5 minutes and then dispersed in N-Methyl-2-pyrrolidone and coated onto Al foil. Electrochemical test cells (Swagelok-type) were assembled in an argon-filled glove box with the coated Al disk as working electrode, lithium metal foil as the counter/reference electrode, and 1 M solution of LiPF_6_ in a 1:1 vol/vol mixture of ethylene carbonate and diethyl carbonate as the electrolyte. Glass microfiber filter paper was used as separator. For electrochemical battery test, the cells were charged and discharged galvanostatically within a fixed voltage window between 2.5 V and 4.0 V (with identical charge and discharge rates). Cyclic Voltammetry (CV) was performed in the same potential window at a scan rate between 5 mV/s to 0.1 mV/s. All electrochemical measurements were performed with Biologic VMP3 potentiostat/galvanostat.

## Additional Information

**How to cite this article**: Cabán-Huertas, Z. *et al.* Aqueous synthesis of LiFePO_4_ with Fractal Granularity. *Sci. Rep.* **6**, 27024; doi: 10.1038/srep27024 (2016).

## Supplementary Material

Supplementary Information

## Figures and Tables

**Table 1:** Root mean square (rms) roughness and scan size of the LiFePO_4_ and LiFePO_4_/C electrode determined from AFM images (Fig. 5).

Specimens	Fractal Dimension obtained by AFM	Rms (nm)
LiFePO_4_	2.38	258.0
LiFePO_4_/C	2.01	207.7
