# Supplementary material for: Aqueous synthesis of LiFePO4 with Fractal Granularity
Source: Sci Rep. 2016 Jun 3;6:27024. doi: 10.1038/srep27024 (PMC4891732; doi:10.1038/srep27024)
Supplement: Supplementary Information [file srep27024-s1.doc]

Supporting information

Aqueous synthesis of LiFePO_4_ with Fractal Granularity.

*Zahilia Caban-Huertas^1^, Omar Ayyad^2^, Deepak P. Dubal^1^, Pedro Gomez-Romero^1^**

^1^Catalan Institute of Nanoscience and Nanotechnology (ICN2), CSIC and The Barcelona Institute of Science and Technology, Campus UAB, Bellaterra, 08193 Barcelona, Spain

^2^Present address: Faculty of Engineering, Dept. of Materials Engineering, Al-Quds University, P.O. Box 20002, East Jerusalem, Palestine.

**Supporting information S1**


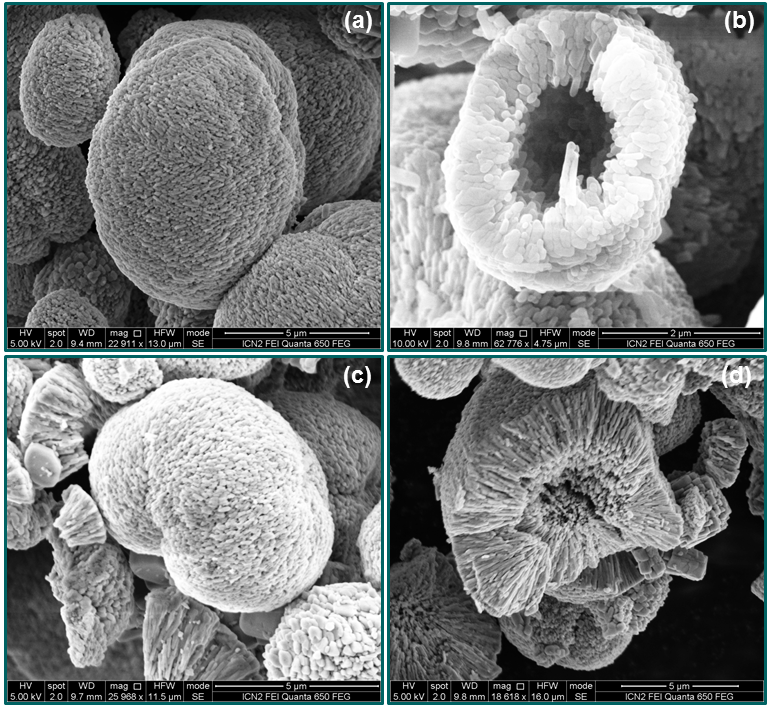


**Figure S1** SEM images of (a,d) LiFePO_4_ and (b,c) LiFePO_4_/C samples, respectively

**Supporting information S2**


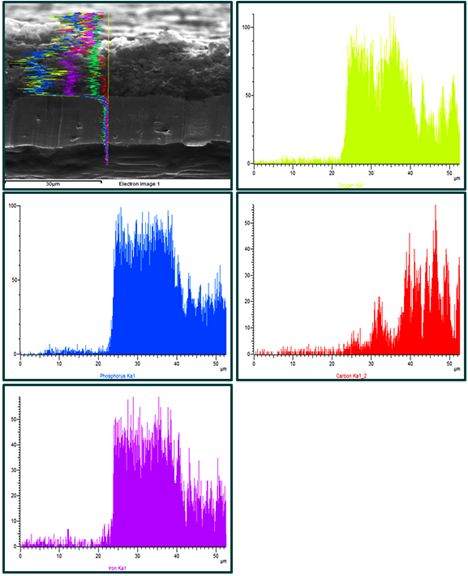


**Figure S2** SEM images of the (a)LiFePO_4_ electrode and element lining analysis.

**Supporting information S3**


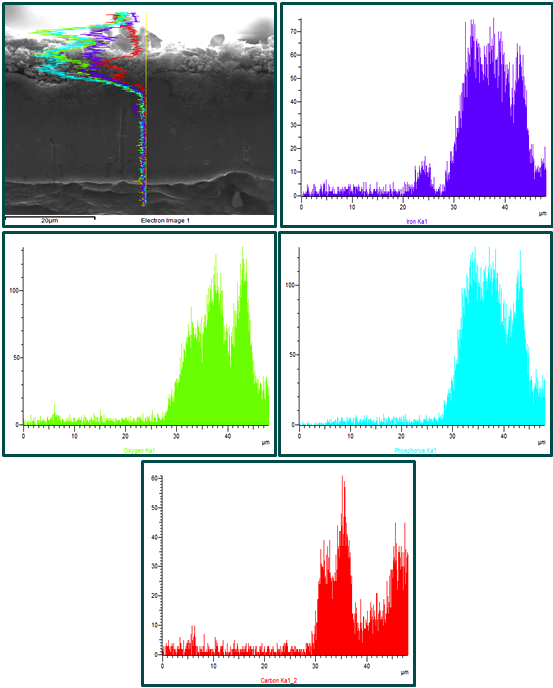


Figure S3. LiFePO_4_/C electrode and element lining analysis.

Cross-sectional EDS analyses were carried out for LiFePO_4_ and LiFePO_4_/C electrodes deposited on Al substrates. The approximate cross-sectional atomic distribution of the C/LiFePO_4_ was determined from EDS elements distribution from the top to the bottom of the film. Li cannot be detected by this method due to its low atomic weight. The elements O, P, Fe and C were quite regularly distributed over the surface of Al in both samples.

**Supporting information S4**

Note on the use of The Randles–Sevcik equation

The evaluation of difussion coefficients for Li ion battery electrodes is frequently made by using the classical Randles–Sevcik equation

$Ip=(2.69x10^{5})n^{\frac{3}{2}}aDCv^{\frac{1}{2}}$(1)

This is not always the correct approach, especially for complex microstructures. Indeed it is a common mistake in the LIB literature to use that equation overlooking the strong assumptions made to derive it,[^1^](#_ENREF_1) namely:

1. Diffusion is one-dimensional from bulk solution towards electrode surface.

2. Electrode surface is ideally smooth.

In lithium battery systems and generally in solid-state electrochemistry diffusion of electroactive species occurs inside the films[^2^](#_ENREF_2). It is known that diffusion inside the electroactive film is no longer one-dimensional; instead a complicated three-dimensional diffusion towards accessible active sites for insertion/extraction processes takes place[^3^](#_ENREF_3).

Considering the second assumption (smoothness) complex microstructures and in particular fractal ones do have significantly rough surfaces. For rough surfaces, the Randles–Sevcik equation should be modified with new parameters as given in equation (4) in the main text, as proposed by Granqvist and col.[^4^](#_ENREF_4)

1. Bard, A.J. & Faulkner, L.R. Electrochemical Methods: Fundamentals and Applications (Wiley, New York, 2001).

2. Pajkossy, T. & Nyikos, L. Diffusion to fractal surfaces—III. Linear sweep and cyclic voltammograms. *Electrochimica Acta* **34**, 181-186 (1989).

3. Pajkossy, T. & Nyikos, L. Diffusion to fractal surfaces—II. Verification of theory. *Electrochimica Acta* **34**, 171-179 (1989).

4. Stromme, M., Niklasson, G.A. & Granqvist, G.C. Determination of fractal dimension by cyclic I-V studies: The Laplace-transform method. *Physical review. B, Condensed matter* **52**, 14192-14197 (1995).
